# CITED2: a novel hub gene downregulated in Hashimoto’s thyroiditis and associated with M1 macrophages via bioinformatics analysis and clinical validation

**DOI:** 10.3389/fimmu.2026.1764100

**Published:** 2026-02-25

**Authors:** Xuefeng Bai, Honghong Duan, Yajing Xu, Haihong Shi, Huibin Huang

**Affiliations:** 1 Department of Endocrinology, the Second Affiliated Hospital of Fujian Medical University, Quanzhou, Fujian, China; 2 Department of Obstetrics and Gynecology, the Second Affiliated Hospital of Fujian Medical University, Quanzhou, Fujian, China; 3 Department of Thyroid Surgery, the Second Affiliated Hospital of Fujian Medical University, Quanzhou, Fujian, China

**Keywords:** CITED2, Hashimoto’s thyroiditis, immune infiltration, M1 macrophage, transcriptome

## Abstract

**Objective:**

This study aims to define the core transcriptomic signatures of thyroid tissue damage in Hashimoto’s thyroiditis (HT), with a specific focus on identifying regulatory hub genes critical for macrophage polarization.

**Methods:**

We performed an integrated analysis of bulk RNA-seq data from a public dataset (GEO: GSE165724), comparing thyroid tissues from HT patients with those from normal controls. The bioinformatic analysis included differential gene expression analysis, functional enrichment analysis, protein-protein interaction (PPI) network construction, and immune cell infiltration profiling using CIBERSORT. Key findings were experimentally validated in an independent clinical cohort (n=32) using quantitative real-time polymerase chain reaction (qRT-PCR), Western blotting, and immunohistochemistry (IHC).

**Results:**

Our analysis identified a core set of 1,752 HT-specific unique differentially expressed genes (DEGs). Network analysis distilled these to 5 up-regulated (e.g., IFNG, CD4, PTPRC) and 10 down-regulated hub genes (e.g., CITED2, TXN2, FOXA2). The immune landscape in HT tissues was markedly remodeled, featuring a significant increase in M1 macrophages. Correlation analysis revealed that IFNG mRNA positively correlated with M1 abundance, whereas CITED2 mRNA showed a strong negative correlation. Clinical validation confirmed significant IFNG upregulation and CITED2 downregulation at the mRNA level. At the protein level, validation demonstrated a significant suppression of CITED2 in HT tissues. IHC co-localization analysis specifically indicated markedly weakened CITED2 expression in the cytoplasm and nucleus of thyroid follicular cells (TFCs) from HT patients. Furthermore, M1 macrophage markers (CD80/CD86) were significantly elevated and positively correlated with autoantibody levels and IFNG mRNA expression, but inversely correlated with CITED2.

**Conclusions:**

This study defines a robust transcriptomic and immune signature for HT. The significant downregulation of CITED2, specifically within thyroid follicular cells, and its inverse correlation with M1 macrophages, imply that its suppression may disrupt thyroid follicular cell function and contribute to the pro-inflammatory immune microenvironment, thereby revealing a novel potential mechanism in HT pathogenesis.

## Introduction

Hashimoto’s thyroiditis (HT) is a common organ-specific autoimmune disorder and the leading cause of hypothyroidism. It is characterized by dense lymphoid infiltration—including lymphocytes, plasma cells, macrophages, and dendritic cells (DCs)—into the thyroid gland, along with elevated serum levels of anti-thyroid peroxidase antibody (TPOAb) and anti-thyroglobulin antibody (TgAb). The global prevalence of HT is influenced by socioeconomic, environmental, and genetic factors, with an estimated prevalence of approximately 4.8%–25.8% in women and 0.9%–7.9% in men (1, 2). Additionally, HT is linked to increased risks of cardiovascular disease (3), malignancy (4), and pregnancy-related complications (5). The pathogenesis of HT involves genetic susceptibility, environmental triggers, and epigenetic dysregulation (6–8), which together disrupt immune homeostasis in the thyroid microenvironment.

In the process of thyroid immune microenvironment disruption, the initiation of the innate immune system involves multiple cell types. Among these, thyroid follicular cells (TFCs) have recently been recognized not merely as passive targets of autoimmune attack, but as active participants in inflammation. By sensing pathogen-associated molecular patterns (PAMPs) and damage-associated molecular patterns (DAMPs) through pattern recognition receptors (PRRs) such as Toll-like receptors (TLRs), TFCs can actively release inflammatory mediators and initiate local innate immune responses (9), thereby potentially priming the subsequent recruitment and activation of immune cells. Among the innate immune cells involved, dysregulated macrophage polarization plays a pivotal role. Studies indicate that excessive iodine intake promotes a shift toward the pro-inflammatory M1 phenotype via enhanced hexokinase 3 (HK3) expression (10). Furthermore, single-cell RNA sequencing has identified a distinct inflammatory macrophage subset in HT characterized by high interleukin-1β (IL-1β) expression, which together with DCs shapes a localized pro-inflammatory milieu (11). Although the precise mechanisms remain incompletely understood, TFCs may influence macrophage polarization via secreted factors or direct cellular interactions.

Moreover, a cytokine network driven by interleukin-17 (IL-17), interleukin-21 (IL-21), and inflammasomes (e.g., NOD-, LRR- and pyrin domain-containing protein 3 (NLRP3)) not only amplifies local inflammation but also directly damages follicular cells through processes such as pyroptosis (12–14). Thyroid stromal cells, such as C–C motif chemokine ligand 21-positive (CCL21^+^) fibroblasts, further contribute by promoting lymphocyte migration and the formation of tertiary lymphoid structures (11). This TFC-driven inflammatory microenvironment ultimately leads to pathological damage primarily mediated by adaptive immune dysregulation (15, 16), including T helper 1 (Th1) cell-mediated apoptosis coupled with regulatory T (Treg) cell functional deficiency (17), T helper 17 (Th17) cell hyperactivation (18), and cytotoxic CD8^+^ T cell activity (19). Nevertheless, the precise molecular mechanisms by which TFCs initiate and sustain this cascade—particularly their interplay with macrophage polarization—remain a crucial unresolved question requiring in-depth investigation.

This study aimed to systematically decipher the transcriptomic reprogramming of thyroid follicular cells in Hashimoto’s thyroiditis and its role in driving the immunodestructive process. Current knowledge has not yet fully elucidated the core transcriptional regulatory network through which TFCs actively initiate and maintain tissue-specific immune injury. We hypothesize that a set of hub genes plays a key role in HT pathogenesis by regulating the crosstalk between TFCs and immune cells, particularly M1 macrophages. To test this hypothesis, we employed an integrated strategy combining bioinformatics analysis with experimental validation. Our approach involved screening differentially expressed genes from transcriptomic data, constructing interaction networks to identify key hubs, analyzing their correlation with immune microenvironment features, and subsequently validating the findings in independent clinical samples. We sought to uncover the core molecular mechanisms by which TFCs mediate immune destruction, offering novel insights into the pathogenesis of HT.

## Methods

### Data sources and clinical cohorts

Bulk RNA sequencing (RNA-seq) data (accession: GSE165724, platform: Illumina HiSeq 2500) were obtained from the Gene Expression Omnibus (GEO). The dataset included normal thyroid tissues (NT, n=12) from patients with laryngeal cancer and tumor-adjacent normal tissues (NAT, n=46) from patients with papillary thyroid carcinoma. Among the NAT samples, 21 exhibited lymphocytic thyroiditis (NAT/LT^+^) while 25 did not (NAT/LT^-^). Following quality control (requiring >6G clean bases per sample, Q30 >85%, appropriate GC content, and adapter trimming), 36 high-quality samples were retained for analysis: NT (n=11), NAT/LT^+^ (n=12), and NAT/LT^-^ (n=13).

For experimental validation, adjacent normal thyroid tissues (n=32) were obtained from thyroidectomy patients (e.g., thyroid nodular hyperplasia, papillary thyroid carcinoma) and cryopreserved. Based on comprehensive clinical and pathological criteria, the samples were stratified into two groups:

- Validation HT (V-HT, n=17): Defined by elevated serum TPOAb and/or TgAb levels, heterogeneous (grillage-pattern) ultrasonographic findings, and histologically confirmed lymphocytic infiltration.
- Validation normal control (V-NC, n=15): Defined by normal serum TPOAb/TgAb levels, homogeneous ultrasonography, and absence of lymphocytic infiltration.

This study was approved by the Institutional Review Board of the Second Affiliated Hospital of Fujian Medical University and was conducted in compliance with the ethical principles for medical research involving human subjects.

This study was approved by the Institutional Review Board of the Second Affiliated Hospital of Fujian Medical University and was conducted in compliance with the ethical principles for medical research involving human subjects.

### Bioinformatic analysis

Genes with low expression (defined as fragments per kilobase of transcript per million mapped reads (FPKM) <1 in over 75% of samples) were filtered out. Differential expression analysis was performed between the NAT/LT^+^ versus NT groups and the NAT/LT^-^ versus NT groups using three independent algorithms: limma, edgeR, and DESeq2. Genes with an adjusted p-value < 0.05 and an absolute fold change |FC| > 2 were considered differentially expressed genes (DEGs). The intersection of DEGs identified by all three methods was defined as the NAT/LT^+^-specific DEGs (comparison: NAT/LT^+^ vs NT) and the NAT/LT^-^-specific DEGs (comparison: NAT/LT^-^ vs NT). The final set of HT-specific unique DEGs was obtained by subtracting the NAT/LT^-^-specific DEGs from the NAT/LT^+^-specific DEGs.

Functional enrichment analysis of these unique DEGs for Gene Ontology (GO) terms and Kyoto Encyclopedia of Genes and Genomes (KEGG) pathways was conducted using the ClusterProfiler R package, with terms/pathways meeting a significance threshold of p < 0.05 considered enriched. Protein-protein interaction (PPI) networks were constructed using the STRING database and visualized in Cytoscape. Key hub genes were identified from the PPI network by applying four topological analysis algorithms (EPC, Closeness, Betweenness, Degree) via the CytoHubba plugin.

Immune cell infiltration abundances in the samples were estimated using CIBERSORT. The deconvolution p-value was calculated for each sample to evaluate reliability, with lower p-values reflecting more significant immune infiltration. Differences in immune cell proportions among the NT, NAT/LT^-^, and NAT/LT^+^ groups were compared using the Kruskal-Wallis test, followed by Dunn’s test with Benjamini–Hochberg false discovery rate (FDR) correction for pairwise comparisons. Spearman’s rank correlation coefficient was calculated to assess the relationships between the expression levels of identified hub genes and the estimated proportions of immune cell types.

### Experimental validation

Total RNA was extracted from clinical specimens using TRIzol reagent, reverse-transcribed, and subjected to reverse transcription−quantitative polymerase chain reaction (RT−qPCR) with gene−specific primers (**Supplementary Table 1**). For Western blot (WB) analysis, proteins were extracted, separated by sodium dodecyl sulphate–polyacrylamide gel electrophoresis (SDS−PAGE), and transferred to polyvinylidene difluoride (PVDF) membranes. Membranes were incubated with an anti−CITED2 antibody (EPR27399−62 [ab314757], Abcam) at a dilution of 1:1000. Immunohistochemistry (IHC) was performed on paraffin−embedded sections using the same antibody at a dilution of 1:400. Antigen retrieval was carried out with Tris−EDTA buffer (pH 9.0), and detection was achieved with 3,3′-diaminobenzidine (DAB). For semi−quantitative analysis, three random non−overlapping high−power fields (400× magnification) per sample were imaged. Positive cells were identified and counted using ImageJ software (National Institutes of Health) based on standardized color thresholds for DAB staining. The positive cell density (cells/mm²) for each field was calculated by dividing the positive cell count by the calibrated field area. The average density from three fields per sample was used for inter−group comparison.

### Statistical analysis

Data were analyzed with GraphPad Prism 8.0.2 and SPSS 23.0. The normality of data distribution and homogeneity of variances were assessed using the Shapiro–Wilk test and F-test, respectively. For comparisons between two groups, the unpaired t-test, Welch’s t-test, or Mann–Whitney U test was used as appropriate. A two−tailed p−value < 0.05 was considered statistically significant.

## Results

**Figure 1:**
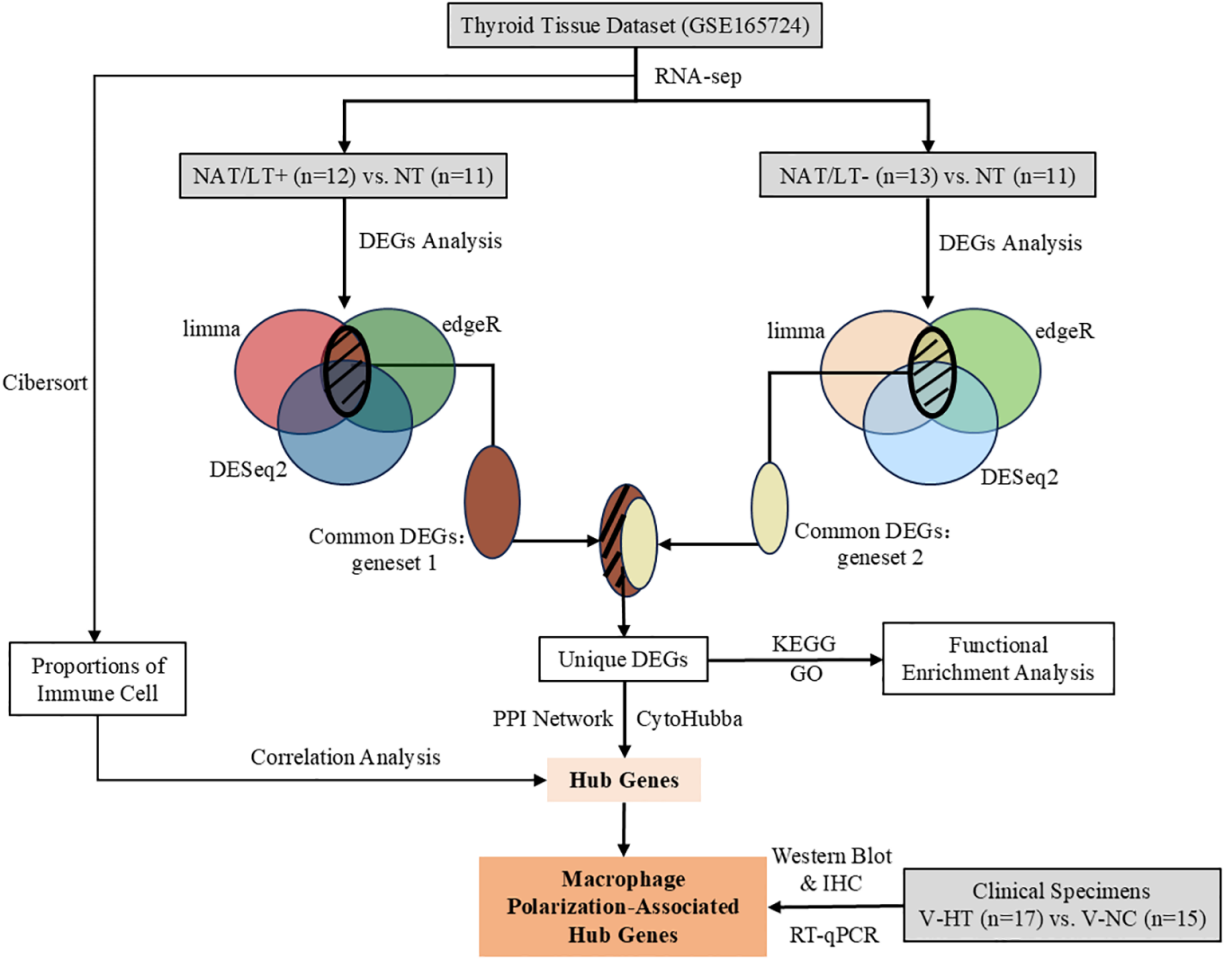
Study flowchart. NAT/LT^+^, tumor−adjacent tissue with lymphocytic thyroiditis; NAT/LT^-^, tumor−adjacent tissue without lymphocytic thyroiditis; NT, normal thyroid; V−NC, validation normal control; V−HT, validation Hashimoto’s thyroiditis; DEGs, differentially expressed genes; PPI, protein–protein interaction network.

We analyzed bulk RNA−seq data (GSE165724) and validated the findings using independent clinical specimens. The overall study design is summarized in **Figure 1**.

### Gene expression profiling in hashimoto’s thyroiditis

**Figure 2:**
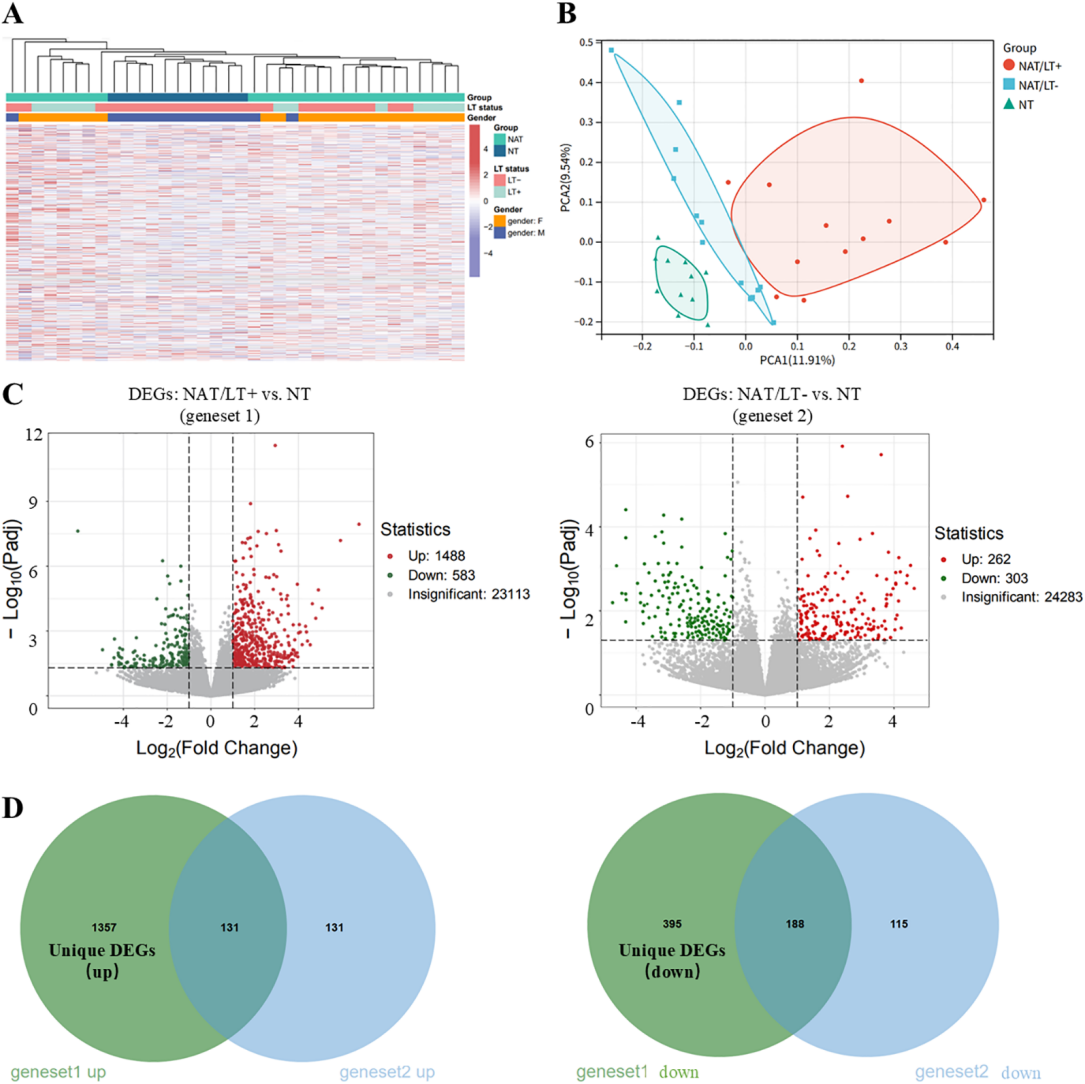
Identification of HT−specific transcriptomic signatures (accession: GSE165724, platform: Illumina HiSeq 2500). **(A)** Hierarchical clustering of 36 thyroid tissue samples based on transcriptomic profiles (Euclidean distance, average linkage). Sample labels indicate ID, sex, and LT status. **(B)** Principal component analysis (PCA) of the transcriptomes. NAT/LT^+^ group, n=12; NAT/LT^-^ group, n=13; NT group, n=11. **(C)** Numbers of differentially expressed genes (DEGs) identified in the comparisons NAT/LT^+^ vs. NT (geneset1) and NAT/LT^-^ vs. NT (geneset2). **(D)** Venn diagrams showing the up− and down−regulated unique DEGs specific to HT, defined as genes present in geneset1 but absent in geneset2.

After filtering low−expression genes, 25,184 genes were retained for subsequent analysis. Unsupervised clustering separated NAT/LT^+^ and NAT/LT^-^ samples from NT samples (**Figure 2A**), and principal component analysis (PCA) confirmed distinct transcriptomic profiles among the groups (**Figure 2B**). Differential expression analysis identified 2,071 DEGs between NAT/LT^+^ and NT (geneset1; 1,488 up−regulated, 583 down−regulated; **Figure 2C**), and 565 DEGs between NAT/LT^-^ and NT (geneset2; 262 up−regulated, 303 down−regulated; **Figure 2C**). After removing common DEGs, 1,752 HT−specific unique DEGs (1,357 up−regulated, 395 down−regulated) were obtained, representing a core molecular signature associated with thyroid immune injury (**Figure 2D**).

### Functional enrichment analysis of HT−specific DEGs

**Figure 3:**
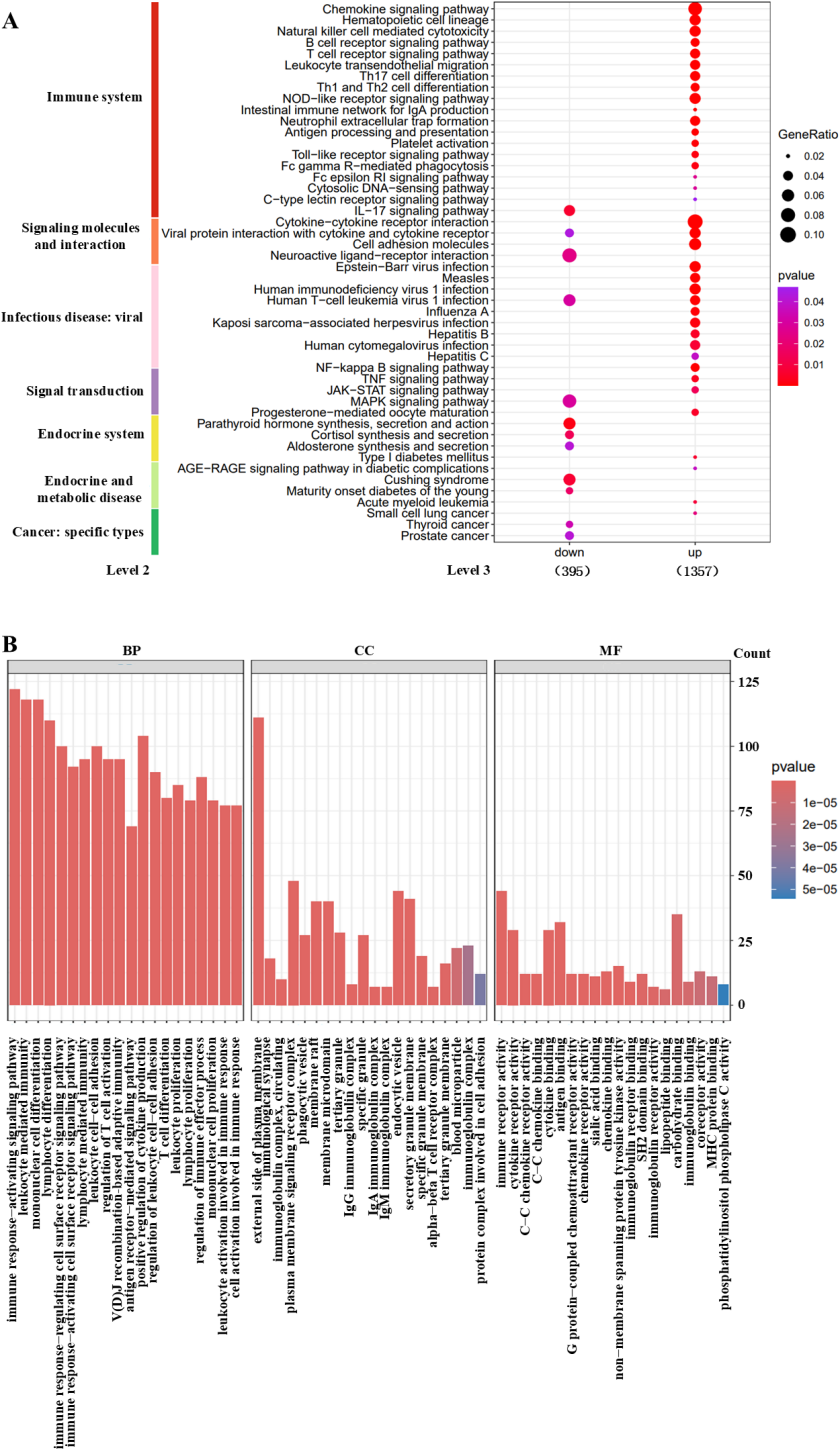
Functional enrichment analysis of HT−specific unique DEGs. **(A)** KEGG pathway enrichment results for up− and down−regulated DEGs. **(B)** Gene Ontology (GO) enrichment terms classified into molecular function, cellular component, and biological process categories.

KEGG pathway analysis revealed distinct enrichment patterns for the up− and down−regulated HT−specific DEGs (**Figure 3A**). Up−regulated genes were significantly enriched in immune− and inflammation−related pathways, including the chemokine signaling pathway, Th1/Th17 cell differentiation, B−cell and T−cell receptor signaling, as well as inflammatory cascades such as nuclear factor kappa B (NF−κB), Janus kinase-signal transducer and activator of transcription (JAK−STAT), and tumor necrosis factor (TNF) signaling pathways. Conversely, down−regulated genes were primarily involved in the IL−17 signaling pathway, neuroactive ligand–receptor interaction, and the mitogen-activated protein kinase (MAPK) cascade.

GO enrichment analysis further underscored the immune−associated functions of these DEGs (**Figure 3B**). Significantly enriched molecular functions included carbohydrate binding, immune receptor and cytokine receptor activity, and antigen binding. Cellular component terms highlighted associations with the external side of the plasma membrane, plasma membrane signaling receptor complexes, and endocytic vesicles. Biological processes were dominated by immune response activation, leukocyte−mediated immunity, monocyte/lymphocyte differentiation, and positive regulation of cytokine production.

### Identification of hub genes and PPI network construction

**Figure 4:**
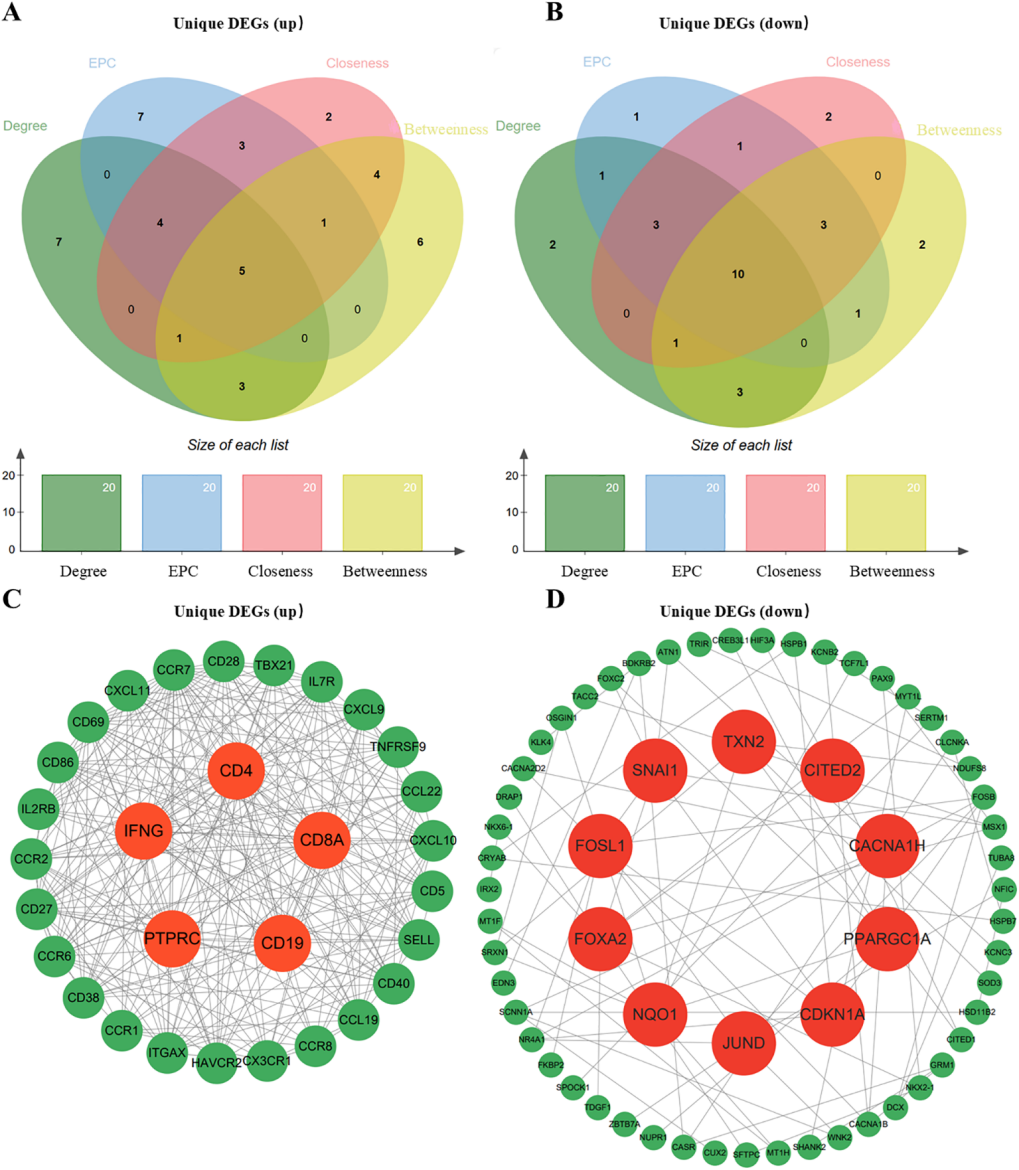
Identification of hub genes from the PPI network. **(A, B)** The top 20 genes ranked by each of four topological algorithms were intersected to identify up−regulated (5 genes) and down−regulated (10 genes) hub genes. **(C)** Subnetwork of up−regulated hub genes (e.g., IFNG, CD4) and their connections. **(D)** Subnetwork of down−regulated hub genes (e.g., CITED2, TXN2) and their associated functions.

A PPI network was constructed from the up−regulated and down−regulated unique DEGs. Based on the intersection of the top 20 genes ranked by four topological algorithms, 5 up−regulated hub genes (IFNG, CD4, CD8A, CD19, PTPRC) and 10 down−regulated hub genes, including CITED2, TXN2, and FOXA2, were identified (**Figures 4A, B**). In the network, up−regulated hubs such as IFNG and CD4 occupied central positions with dense connections to other up−regulated genes (**Figure 4C**). Down−regulated hubs, including CITED2, TXN2, PPARGC1A, and CDKN1A, were associated with functions related to immune activation, oxidative metabolism, and cell−cycle inhibition (**Figure 4D**), indicating their involvement in HT−related immune dysregulation.

### Immune cell infiltration and correlation with hub genes

**Figure 5:**
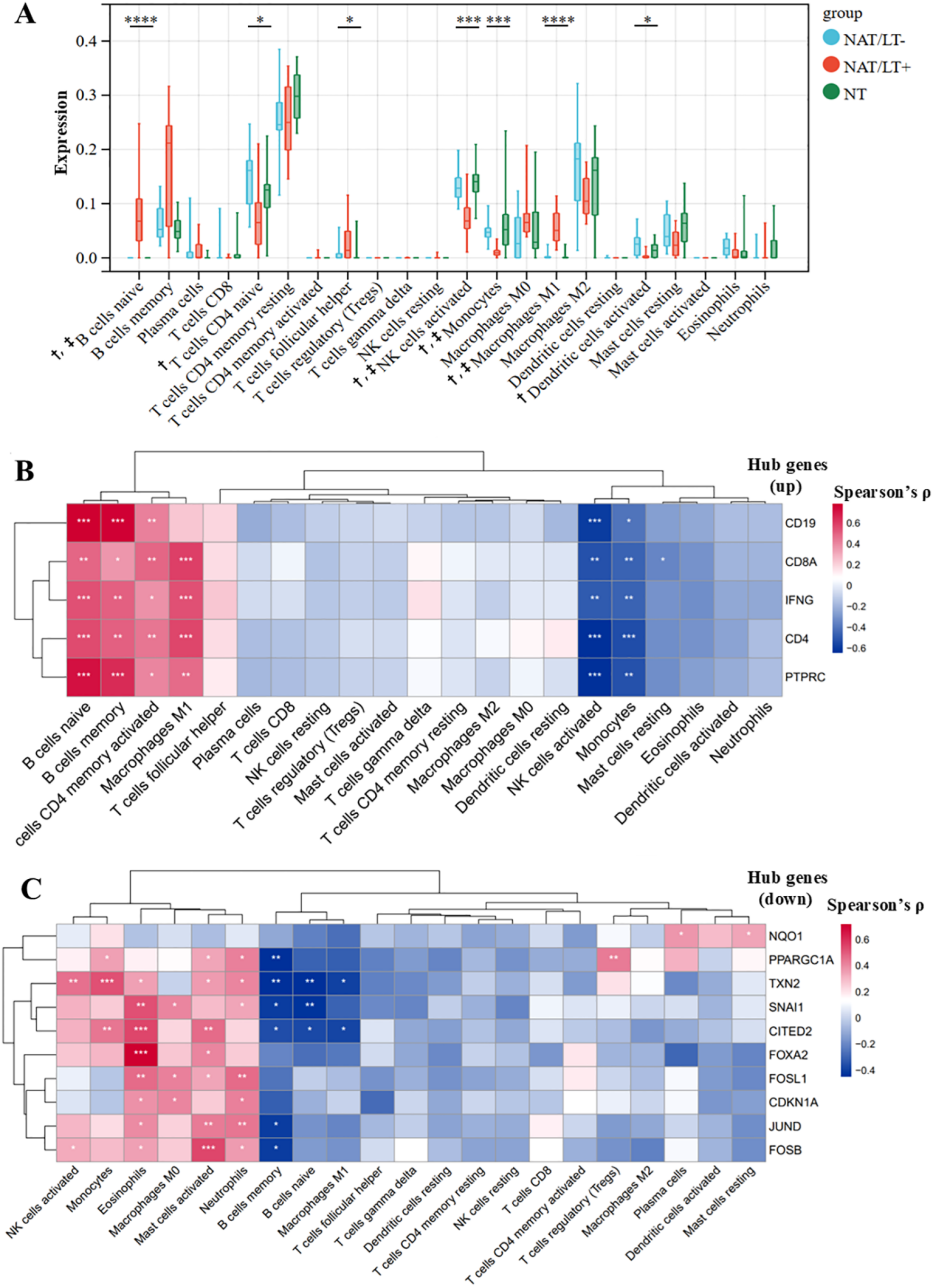
Immune cell infiltration and its correlation with hub−gene expression in Hashimoto’s thyroiditis tissues. **(A)** Relative abundance of immune cells in NAT/LT^+^, NAT/LT^-^, and NT groups, analyzed by CIBERSORT. Differences across groups were assessed using the Kruskal–Wallis test (*p < 0.05, **p < 0.01, ***p < 0.001), followed by Dunn’s test with Benjamini–Hochberg (FDR) correction for pairwise comparisons. Symbols † and ‡ indicate adjusted p < 0.05 for NAT/LT^+^ vs. NAT/LT^-^ and NAT/LT^+^ vs. NT, respectively. Specifically, FDR-corrected pairwise comparisons revealed that within the NAT/LT^+^ group, the proportions of naïve B cells and M1 macrophages were significantly higher than those in both the NAT/LT^-^ and NT groups; the proportions of activated NK cells and monocytes were significantly lower than those in both the NAT/LT^-^ and NT groups; and the proportions of naïve CD4^+^ T cells and activated dendritic cells (DCs) were significantly lower only when compared to the NAT/LT^-^ group. **(B)** Spearman correlations between up−regulated hub genes and immune−cell proportions. **(C)** Spearman correlations between down−regulated hub genes and immune−cell proportions. Significance levels: *p < 0.05, **p < 0.01, ***p < 0.001, ****p < 0.0001.

To characterize the immune microenvironment in HT, we evaluated immune cell infiltration using CIBERSORT. The deconvolution p-value assessment revealed that 10/12 samples in the NAT/LT^+^ group had p < 0.05, indicating robust immune infiltration, whereas 11/13 samples in the NAT/LT^-^ group and all NT samples had p > 0.05. The analysis confirmed a remodeled immune landscape in HT tissues, as illustrated in **Figure 5A**. Significant differences (p< 0.05) in cell proportions were observed among the NT, NAT/LT^-^, and NAT/LT^+^ groups for naïve B cells, naïve CD4^+^ T cells, follicular helper T (Tfh) cells, activated natural killer (NK) cells, monocytes, M1 macrophages, and activated DCs. Specifically, within the NAT/LT^+^ group, naïve B cells and M1 macrophages were significantly increased compared to both NAT/LT^-^ and NT. In contrast, activated NK cells and monocytes were decreased. Naïve CD4^+^ T cells and activated DCs were lower in the NAT/LT^+^ group only when compared to the NAT/LT^-^ group. Although M2 macrophages were more abundant than M1 macrophages across all groups, their levels did not differ significantly.

Spearman correlation analysis was performed to link hub gene expression to immune cell proportions (**Figures 5B, C**). Among the up−regulated hub genes, PTPRC, CD4, CD8A, and IFNG showed positive correlations with M1 macrophages (p < 0.05) but negative correlations with activated NK cells and monocytes; CD19 did not follow this pattern. Among the down−regulated hub genes, TXN2 and CITED2 were negatively correlated with M1 macrophages, while PPARGC1A, TXN2, SNAI1, and CITED2 were negatively correlated with naïve or memory B cells. Conversely, TXN2 and CITED2 were positively correlated with monocytes. These associations suggest that the identified hub genes may influence immune cell polarization and activation, contributing to HT pathogenesis.

### Clinical validation of M1 macrophage-associated hub genes

Building upon the CIBERSORT and correlation analyses that linked M1 macrophage abundance to specific hub genes, we selected the up−regulated gene IFNG and the down−regulated genes TXN2 and CITED2 for validation in independent clinical samples. The expression of canonical macrophage polarization markers was also examined, with CD80 and CD86 representing the M1 phenotype, and CD163 and MRC1 representing the M2 phenotype.

**Figure 6:**
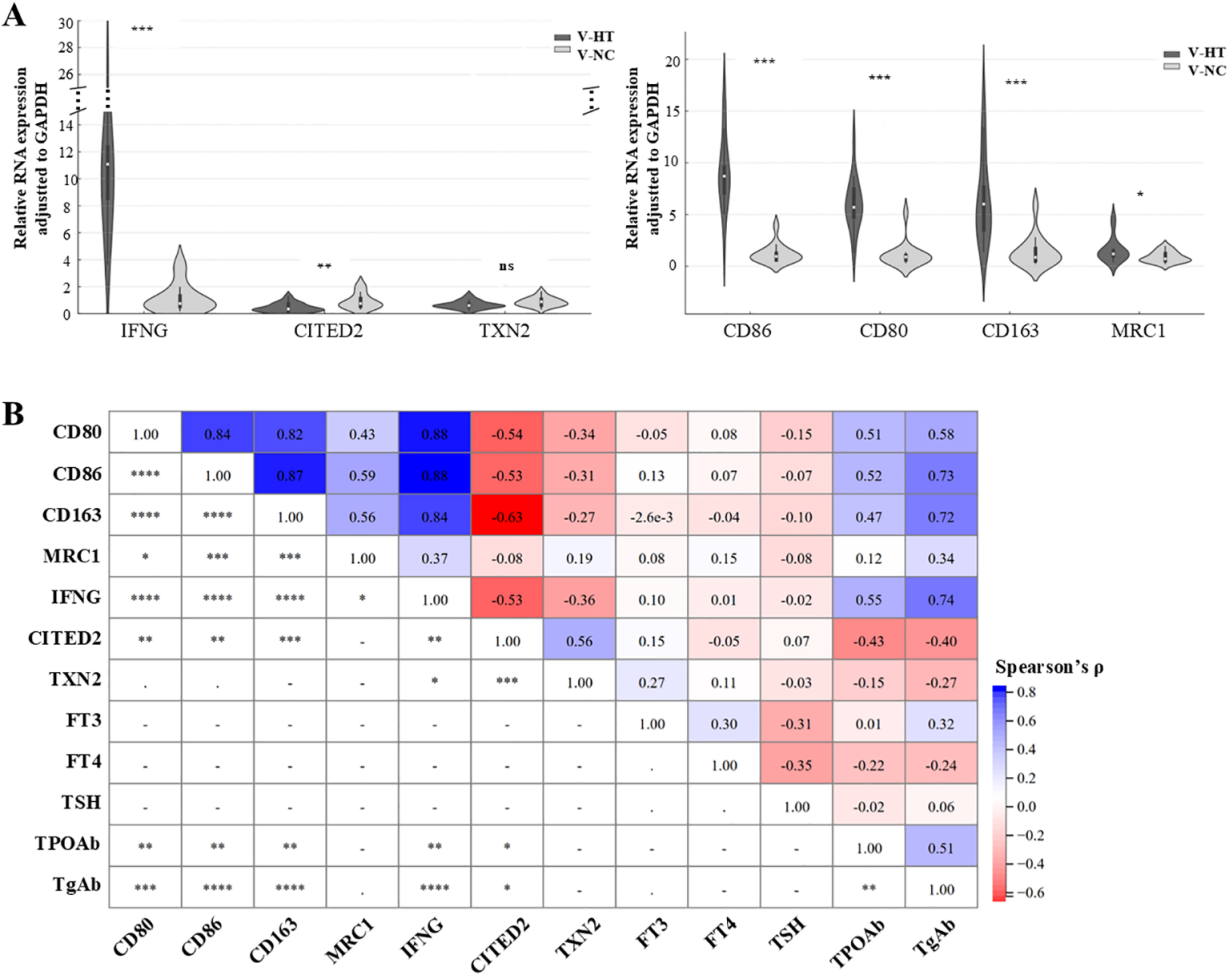
Validation of M1−associated hub genes and macrophage polarization markers. **(A)** RT−qPCR analysis of selected hub genes (IFNG, CITED2, TXN2) and macrophage polarization markers (M1: CD80, CD86; M2: CD163, MRC1) in validation cohorts (V−HT, n=17; V−NC, n=15). Group comparisons were performed using the Mann–Whitney U test (*p < 0.05, **p < 0.01, ***p < 0.001). **(B)** Spearman correlation matrix integrating expression levels of M1−associated hub genes, macrophage markers, and clinical parameters (FT3, FT4, TSH, TPOAb, TgAb) across all validation samples (N = 32). Color scale indicates correlation strength and direction (blue: positive; red: negative). Significance levels: *p < 0.05, **p < 0.01, ***p < 0.001, ****p < 0.0001.

The validation cohort comprised 32 patients who underwent thyroid surgery between December 2023 and July 2024, including 17 with HT (V−HT) and 15 normal controls (V−NC); detailed clinicopathological characteristics are provided in **Supplementary Table 2**. Preoperative thyroid function (FT3, FT4, TSH) did not differ between groups, but TPOAb and TgAb levels were significantly elevated in the V−HT group (p < 0.001; **Table 1**). qPCR analysis confirmed significant up−regulation of IFNG (p < 0.001) and down−regulation of CITED2 (p < 0.01) in V−HT versus V−NC, while TXN2 showed a non−significant decreasing trend (**Figure 6A**). The M1 markers CD80 and CD86 were markedly elevated in V−HT group (both p < 0.001). The M2 markers CD163 (p < 0.001) and MRC1 (p < 0.05) were also differentially expressed between groups.

**Table 1:** Basic information of the study - RT-qPCR (from December 1, 2023 to July 1, 2024).

Parameters	V-NC Group	V-HT Group	P
	(n=15)	(n=17)	
Gender (Male/Female)	2/13	0/17	
Age (years)	45 ± 14	43 ± 11	0.591
FT3 (pmol/L)	4.86 ± 0.39	5.02 ± 0.95	0.547
FT4 (pmol/L)	16.73 ± 2.24	15.93 ± 4.29	0.52
TSH (mIU/L)	1.57 (1.34-3.98)	1.73 (1.1-2.37)	0.723
TPOAb (IU/L)	9.0 (9.0-18.7)	278.0 (138.0-600.0)	<0.001
TgAb (IU/L)	16.50 (15.30-20.50)	457.0 (82.3-728.95)	<0.001
TRAb (IU/L)	0.80 (0.80-1.13)	0.80 (0.80-1.03)	0.755

FT3, Free triiodothyronine; FT4, Free thyroxine; TSH, Thyroid-stimulating hormone; TPOAb, Thyroid peroxidase antibody; TgAb, Thyroglobulin antibody; TRAb, Thyrotropin receptor antibody; V-NC, Validation normal control group; V-HT, Validation Hashimoto’s thyroiditis group.

We next performed correlation analysis between the validated markers and clinical/molecular parameters (**Figure 6B**). The expression of M1 markers CD80 and CD86 showed significant positive correlations with TPOAb, TgAb, and IFNG levels (all p < 0.01), and negative correlations with CITED2 (p < 0.01), but not with TXN2. CD163 expression also correlated positively with TPOAb, TgAb, and IFNG (p < 0.01), and negatively with CITED2 (p < 0.01). In contrast, MRC1 exhibited only a weak positive correlation with IFNG (p< 0.05) and showed no significant association with autoantibodies or the other validated hub genes.

These findings underscore a strong link between M1 macrophage activation and HT severity, which is closely associated with IFNG and CITED2 expression. While interferon−γ (IFN−γ), encoded by IFNG, is a well-established inducer of M1 polarization, our data suggest that CITED2 may function as a novel potential regulator of macrophage dynamics in HT pathogenesis.

### Protein−level validation of CITED2 in HT thyroid tissues

**Figure 7:**
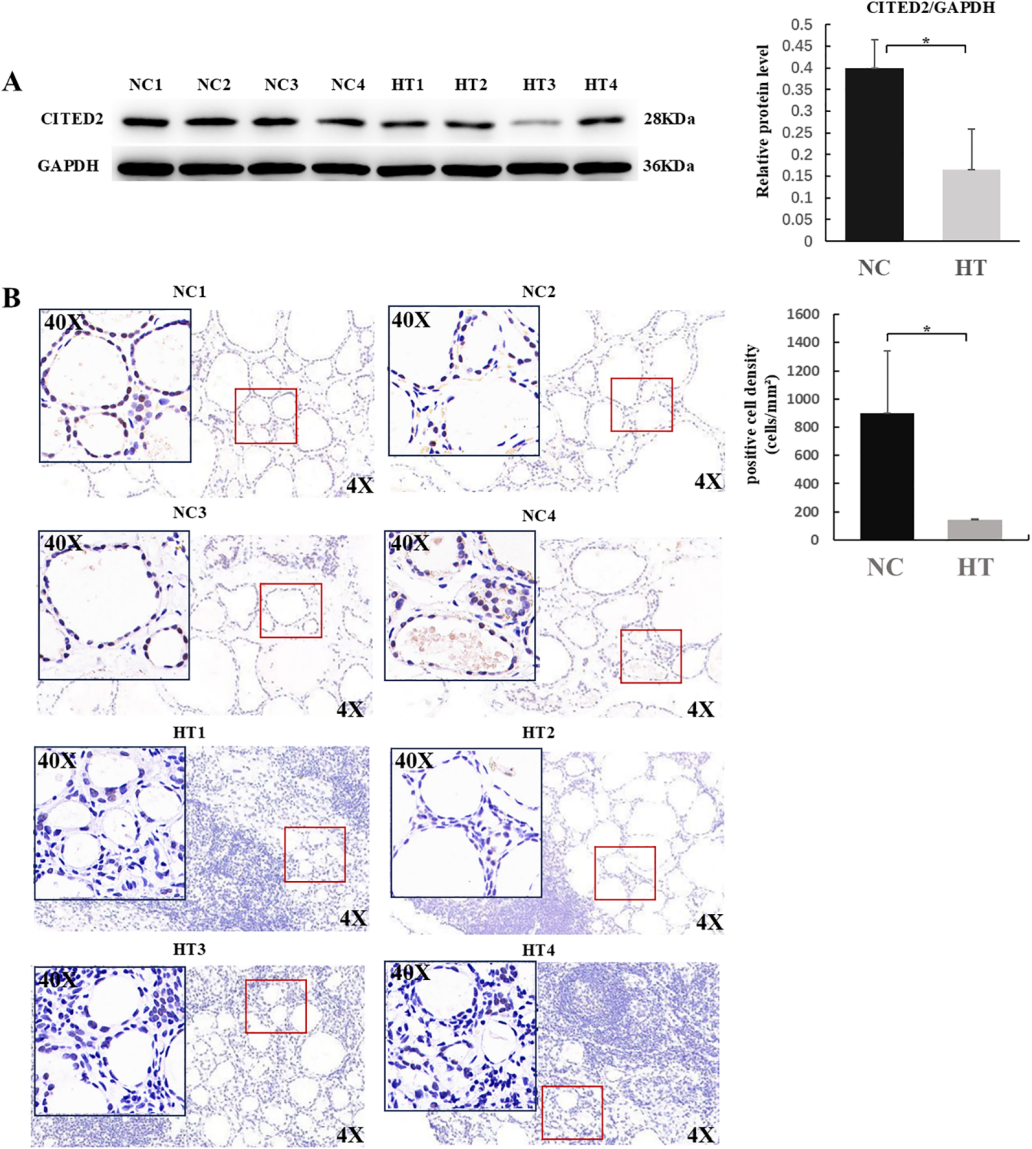
Protein expression and localization of CITED2 in thyroid tissues. **(A)** Western blot analysis of CITED2 protein levels in Hashimoto’s thyroiditis (HT) versus normal control (NC) samples, normalized to GAPDH. **(B)** Representative immunohistochemical staining of CITED2 (brown signal) at 4× and 40× magnification, with semi−quantitative analysis shown as a bar graph (positive cell density, cells/mm²). For semi−quantitative analysis, three random non−overlapping high−power fields (400×) per sample were captured. Positive cells were counted using Image J based on standardized DAB thresholding, and density was calculated per calibrated field area. Data are presented as mean density per sample. HT, n=4; NC, n=4. All samples are from the clinical validation cohort in **Figure 6**. Statistical significance was assessed by the Mann–Whitney U test (*p < 0.05).

To further validate CITED2 expression at the protein level, we analyzed thyroid tissues from four HT patients and four normal controls. Western blot revealed that CITED2 protein levels (normalized to GAPDH) were significantly lower in the HT group compared to controls (p < 0.05; **Figure 7A**). IHC was performed to assess the cellular localization and expression intensity of CITED2. As shown in **Figures 7B**, IHC staining qualitatively demonstrated attenuated CITED2 signal in the cytoplasm and nuclei of thyroid follicular cells in HT tissues. Semi−quantitative analysis based on positive cell density (cells/mm²) confirmed a significant reduction in CITED2 expression in the HT group compared to normal controls (p< 0.05; **Figure 7B**). Together, these results confirm the down−regulation of CITED2 in HT at the protein level, supporting its potential involvement in HT pathogenesis.

## Discussion

Hashimoto’s thyroiditis involves progressive destruction of TFCs and widespread immune infiltration, yet the intercellular regulatory networks remain poorly defined. Our study revealed a disrupted immune microenvironment in HT, characterized by abnormal activation of naïve B cells, Tfh cells, and M1 macrophages, along with up-regulated pro−inflammatory pathways. By integrating transcriptomics with clinical validation, we pinpointed CITED2 as a central hub gene that is consistently down−regulated in HT TFCs at both mRNA and protein levels. This deficiency strongly correlated with increased M1 macrophage infiltration and inversely with serological markers of disease severity (TPOAb, TgAb). Thus, our findings nominate CITED2 not merely as a candidate biomarker, but as a critical molecular nexus potentially bridging intrinsic TFC dysfunction to innate (M1 macrophage−driven) and adaptive immune dysregulation, thereby reinforcing the concept of TFC−initiated immune remodeling in HT pathogenesis.

Notably, our clinical validation demonstrated significant upregulation of both classical M1 (CD80, CD86) and M2 (CD163, MRC1) macrophage markers in HT tissues. This suggests the thyroid microenvironment in HT harbors a mixed population of activated macrophages, aligning with the known plasticity and phenotypic heterogeneity of macrophages in chronic inflammation (20). Critically, however, correlation analysis revealed distinct patterns: M1 markers (CD80/CD86) showed strong positive correlations with serum autoantibodies (TPOAb, TgAb) and IFNG expression, and a negative correlation with CITED2. In contrast, the M2 marker CD163 had weaker associations, and MRC1 showed no significant correlations with these parameters. This pattern underscores that the M1−like macrophage subset likely holds higher pathological relevance in driving HT immunopathology. M1 macrophages, known for secreting pro−inflammatory cytokines (e.g., TNF−α, IL−1β) and possessing antigen−presenting capacity, may directly participate in thyroid follicle destruction and amplify autoimmunity (11, 12). The concomitant increase in M2 cells may represent a compensatory regulatory or repair response, but their function in HT appears insufficient to counterbalance the inflammatory drive.

The down−regulation of CITED2 showed a distinct immune correlation profile, being negatively associated with M1 macrophages, naïve B cells, and memory B cells, but positively with monocytes and eosinophils. This complex signature raises key mechanistic questions: Does CITED2 modulate immune cells indirectly via paracrine signals from TFCs? Does its loss directly alter the immunogenic profile of TFCs? Could CITED2 contribute to immune dysregulation by rewiring TFC metabolism?

The capacity of CITED2 to link TFC dysfunction with immune dysregulation may stem from its evolutionarily conserved role as a transcriptional modulator. It fine−tunes gene expression by dynamically regulating the coactivators CREB−binding protein (CBP) and p300 (21, 22). Context dictates its activity: during development, CITED2 acts as a coactivator for factors such as transcription factor AP−2 (TFAP2) and suppressor of mothers against decapentaplegic homolog 2/3 (SMAD2/3) (23, 24), whereas in inflammatory settings it exerts potent transcriptional repression by competitively inhibiting NF−κB, hypoxia−inducible factor 1−alpha (HIF−1α), signal transducer and activator of transcription 1 (STAT1), and interferon regulatory factor 1 (IRF1) pathways (25–28). This anti−inflammatory role is supported by evidence that myeloid−specific Cited2knockout mice show increased susceptibility to endotoxin shock and atherosclerosis (27, 28). Therefore, CITED2 down−regulation in HT likely releases a “molecular brake” on pro−inflammatory pathways such as NF−κB and STAT1/IRF1, potentially creating a permissive environment for M1 macrophage polarization and subsequent immune activation.

Building on this molecular framework and our correlative findings, we propose several hypothetical yet plausible mechanistic models through which CITED2 deficiency could drive HT immunopathology. These models, which remain to be experimentally validated, aim to connect the observed CITED2 down-regulation with established pathogenic features of HT:

First, we speculate that CITED2−deficient TFCs may secrete specific chemokines that recruit and polarize monocytes toward an M1 phenotype. Chemokines such as C−X−C motif chemokine ligand 9 (CXCL9), CXCL10, and C−C motif chemokine ligand 2 (CCL2) are elevated in HT and correlate with disease activity (29, 30). As a known inhibitor of NF−κB and STAT1/IRF1 (25, 28), it is plausible that reduced CITED2 could enhance activation of these pathways, thereby elevating the expression of chemokines such as CXCL10. If experimentally confirmed in TFCs, this mechanism could establish a chemokine-driven loop that recruits monocytes and, in concert with local IFN−γ and TNF−α, promotes their M1 polarization, potentially sustaining an inflammatory microenvironment.

Second, down−regulation of CITED2 might directly remodel the immune−related gene network in TFCs. A testable hypothesis is that this could confer TFCs with aberrant antigen-presenting capacity. Spatial transcriptomics has shown elevated CD74 and macrophage migration inhibitory factor (MIF) expression in HT TFCs, colocalizing with immune activation (31). CITED2 may influence major histocompatibility complex class II (MHC−II) expression; thus, its loss might be permissive for the aberrant HLA−DR display on TFCs observed in HT—enabling autoantigen presentation to CD4^+^ T cells (19, 32). Concurrently, increased MIF might engage herpesvirus entry mediator (HVEM) to activate NF−κB and promote Th17 differentiation (33, 34), linking TFC−intrinsic changes to adaptive immune imbalance.

Third, given CITED2’s emerging role in metabolic regulation, its deficiency might induce “metabolic–immune” dysregulation in TFCs. Based on reports that Th1 cytokines can suppress sirtuin 1 (SIRT1), up−regulating HIF−1α and vascular endothelial growth factor A (VEGF−A) in TFCs (35). Since CITED2 antagonizes HIF−1α, its loss could trigger a similar metabolic shift, potentially creating a feed−forward loop that amplifies inflammatory and immune responses.

While these models implicate CITED2 deficiency in promoting immune dysregulation, they also highlight broader gaps in our understanding. First, the specific gene networks controlled by CITED2 in TFCs remain poorly defined. Beyond known inflammatory pathways, does it also regulate TFC homeostasis via peroxisome proliferator−activated receptor gamma (PPAR−γ) (36) or metabolic reprogramming akin to its role in hepatic gluconeogenesis (37)? Second, the upstream drivers of CITED2 down−regulation in HT are unknown. Potential contributors include genetic susceptibility, epigenetic modifications, or feedback inhibition by local inflammatory cytokines (e.g., IFN−γ, TNF−α). Third, CITED2 function exhibits marked cell−type specificity, acting as a metastasis promoter in some cancers (38–41) while being essential for hematopoietic stem cell homeostasis (42). Resolving these questions is crucial for fully elucidating CITED2’s role in HT.

Several limitations of this study should be considered. First, the immune cell proportions provided by CIBERSORT are bioinformatic estimates based on gene expression signatures, not direct cellular counts. Second, the discovery analysis relied on a public dataset where control tissues were from patients with laryngeal cancer, and tissues of interest were adjacent to PTC. Although we rigorously subgrouped samples based on lymphocytic thyroiditis, the potential influence of a PTC background is a recognized confounder. To address this, we established an independent validation cohort comprising patients definitively diagnosed with HT based on stringent serological, ultrasonographic, and histopathological criteria. The successful validation of CITED2 downregulation and its associations in this cohort strengthens the link of our findings to HT pathogenesis.

In summary, this study identifies CITED2 as a central molecular node linking TFC dysfunction to immune microenvironment dysregulation in HT. Future investigations should prioritize (1): using TFC−specific models to define CITED2’s cell−autonomous and paracrine effects on immune cells, testing the chemokine- and antigen-presentation-related hypotheses (2); employing genome−wide approaches to map its downstream target genes and upstream regulatory networks in TFCs; and (3) dissecting its potential role as a “metabolic–immune” integrator in TFCs. These efforts will clarify how follicular cell defects sustain immune pathology in HT and may reveal novel therapeutic targets.

## Data Availability

The datasets presented in this study can be found in online repositories. The names of the repository/repositories and accession number(s) can be found below: https://www.ncbi.nlm.nih.gov/, GSE165724.
