# Supplementary material for: CITED2: a novel hub gene downregulated in Hashimoto’s thyroiditis and associated with M1 macrophages via bioinformatics analysis and clinical validation
Source: Front Immunol. 2026 Feb 25;17:1764100. doi: 10.3389/fimmu.2026.1764100 (PMC12975973; doi:10.3389/fimmu.2026.1764100)
Supplement: Supplementary file 1 [file Table1.docx]

| Table S1. Primer sequences used for quantitative real-time PCR (qRT-PCR) analysis | |
| --- | --- |
| Gene symbol | Primer sequence (5'→3') |
| GAPDH | F: TGTTGCCATCAATGACCCCTT |
|  | R: CTCCACGACGTACTCAGCG |
| IFNG | F: TCGGTAACTGACTTGAATGTCCA |
|  | R: TCGCTTCCCTGTTTTAGCTGC |
| CITED2 | F: GCCGCCCAATGTCATAGACAC |
|  | R: CAGCTCCTTGATGCGGTCCAAA |
| TXN2 | F: CTGGTGGCCTGACTGTAACAC |
|  | R: TGACCACTCGGTCTTGAAAGT |
| CD86 | F: CTGCTCATCTATACACGGTTACC |
|  | R: GGAAACGTCGTACAGTTCTGTG |
| CD80 | F: GGCCCGAGTACAAGAACCG |
|  | R: TCGTATGTGCCCTCGTCAGAT |
| CD163 | F: TTTGTCAACTTGAGTCCCTTCAC |
|  | R: TCCCGCTACACTTGTTTTCAC |
| MRC1 | F: AGCCAACACCAGCTCCTCAAGA |
|  | R: CAAAACGCTCGCGCATTGTCCA |
| *Primers designed via Primer-BLAST (NCBI). | |
