# Supplementary material for: CITED2: a novel hub gene downregulated in Hashimoto’s thyroiditis and associated with M1 macrophages via bioinformatics analysis and clinical validation
Source: Front Immunol. 2026 Feb 25;17:1764100. doi: 10.3389/fimmu.2026.1764100 (PMC12975973; doi:10.3389/fimmu.2026.1764100)
Supplement: Supplementary file 2 [file Table2.docx]

| Supplementary Table 2: Clinical and Pathological Characteristics of Individual Patients in the Validation Cohort | | | | | | | | | | | | |
| --- | --- | --- | --- | --- | --- | --- | --- | --- | --- | --- | --- | --- |
| Group | Patient  ID | Age (years) | Gender | FT3  (pmol/L) | FT4  (pmol/L) | TSH  (mIU/L) | TPOAb  (IU/L) | TgAb  (IU/L) | TRAb  (IU/L) | Disease State | Tissue Type | Lymphocytic  Thyroiditis Status |
| V-HT | HT1 | 39 | Female | 5.44 | 16.6 | 1.37 | 53.6 | 493 | 0.8 | Follicular Thyroid Neoplasm  (FTN) | Normal appearing thyroid tissue adjacent to FTN | Present |
| V-HT | HT2 | 61 | Female | 3.11 | 11.2 | 2.75 | 24.6 | 457 | 0.8 | Papillary Thyroid Carcinoma  (PTC) | Normal appearing thyroid tissue adjacent to PTC | Present |
| V-HT | HT3 | 34 | Female | 5.93 | 17.6 | 2.13 | 600 | 1088 | 1.88 | Chronic Lymphocytic Thyroiditis | Thyroid parenchyma involved by lymphocytic thyroiditis^†^ | Present |
| V-HT | HT4 | 49 | Female | 5.34 | 15.8 | 1.22 | 166 | 503 | 0.8 | Papillary Thyroid Carcinoma  (PTC) | Normal appearing thyroid tissue adjacent to PTC | Present |
| V-HT | HT5 | 48 | Female | 4.46 | 15.5 | 1.13 | 190 | 366 | 0.877 | Papillary Thyroid Carcinoma  (PTC) | Normal appearing thyroid tissue adjacent to PTC | Present |
| V-HT | HT6 | 24 | Female | 6.75 | 20.2 | 0.005 | 9 | 686 | 0.8 | Papillary Thyroid Carcinoma  (PTC) | Normal appearing thyroid tissue adjacent to PTC | Present |
| V-HT | HT7 | 37 | Female | 5.31 | 18.2 | 2.03 | 335 | 122 | 0.8 | Follicular Thyroid Neoplasm  (FTN) | Normal appearing thyroid tissue adjacent to FTN | Present |
| V-HT | HT8 | 38 | Female | 4.17 | 14.5 | 2.41 | 119 | 524 | 0.8 | Thyroid Nodular Hyperplasia  (TNH) | Normal appearing thyroid tissue adjacent to TNH | Present |
| V-HT | HT9 | 42 | Female | 4.62 | 15.3 | 2.33 | 600 | 927 | 0.963 | Papillary Thyroid Carcinoma  (PTC) | Normal appearing thyroid tissue adjacent to PTC | Present |
| V-HT | HT10 | 50 | Female | 3.68 | 7.32 | 30.1 | 157 | 360 | 1.47 | Papillary Thyroid Carcinoma  (PTC) | Normal appearing thyroid tissue adjacent to PTC | Present |
| V-HT | HT11 | 28 | Female | 4.38 | 17.1 | 0.88 | 278 | 23.2 | 1.1 | Papillary Thyroid Carcinoma  (PTC) | Normal appearing thyroid tissue adjacent to PTC | Present |
| V-HT | HT12 | 43 | Female | 5.11 | 25.1 | 1.6 | 222 | 205 | 0.8 | Papillary Thyroid Carcinoma  (PTC) | Normal appearing thyroid tissue adjacent to PTC | Present |
| V-HT | HT13 | 38 | Female | 5.24 | 12.1 | 1.81 | 600 | 859 | 0.8 | Thyroid Nodular Hyperplasia  (TNH) | Normal appearing thyroid tissue adjacent to TNH | Present |
| V-HT | HT14 | 64 | Female | 5.15 | 19.55 | 1.726 | 1300 | 42.6 | 0.942 | Chronic Lymphocytic Thyroiditis | Thyroid parenchyma involved by lymphocytic thyroiditis^†^ | Present |
| V-HT | HT15 | 54 | Female | 4.73 | 18.4 | 1.01 | 575 | 31.9 | 0.8 | Papillary Thyroid Carcinoma  (PTC) | Normal appearing thyroid tissue adjacent to PTC | Present |
| V-HT | HT16 | 40 | Female | 5.14 | 17.3 | 6.28 | 344 | 32.7 | 0.8 | Papillary Thyroid Carcinoma  (PTC) | Normal appearing thyroid tissue adjacent to PTC | Present |
| V-HT | HT17 | 35 | Male | 6.78 | 9.02 | 1.07 | 600 | 772 | 1.91 | Thyroid Nodular Hyperplasia  (TNH) | Normal appearing thyroid tissue adjacent to TNH | Present |
| V-NC | NC1 | 60 | Female | 5.14 | 14.5 | 1.38 | 11.2 | 17.2 | 0.8 | Thyroid Nodular Hyperplasia  (TNH) | Normal appearing thyroid tissue adjacent to TNH | Absent |
| V-NC | NC2 | 58 | Female | 4.8 | 13.6 | 1.68 | 25.2 | 20.5 | 0.8 | Thyroid Nodular Hyperplasia  (TNH) | Normal appearing thyroid tissue adjacent to TNH | Absent |
| V-NC | NC3 | 14 | Female | 5.86 | 19.3 | 1.56 | 9 | 104 | 0.8 | Thyroid Nodular Hyperplasia  (TNH) | Normal appearing thyroid tissue adjacent to TNH | Absent |
| V-NC | NC4 | 50 | Female | 4.65 | 21.5 | 0.043 | 9 | 14.8 | 0.8 | Papillary Thyroid Carcinoma  (PTC) | Normal appearing thyroid tissue adjacent to PTC | Absent |
| V-NC | NC5 | 30 | Female | 4.61 | 15.9 | 4.45 | 9 | 16 | 0.8 | Papillary Thyroid Carcinoma  (PTC) | Normal appearing thyroid tissue adjacent to PTC | Absent |
| V-NC | NC6 | 39 | Female | 4.59 | 15.6 | 0.232 | 9.68 | 15.3 | 0.8 | Thyroid Nodular Hyperplasia  (TNH) | Normal appearing thyroid tissue adjacent to TNH | Absent |
| V-NC | NC7 | 49 | Male | 5.21 | 14.8 | 2.61 | 9 | 17.7 | 0.8 | Papillary Thyroid Carcinoma  (PTC) | Normal appearing thyroid tissue adjacent to PTC | Absent |
| V-NC | NC8 | 58 | Female | 4.81 | 19.9 | 1.34 | 9 | 15.1 | 0.83 | Papillary Thyroid Carcinoma  (PTC) | Normal appearing thyroid tissue adjacent to PTC | Absent |
| V-NC | NC9 | 38 | Female | 4.21 | 15.4 | 3.98 | 18.7 | 26.8 | 1.23 | Thyroid Nodular Hyperplasia  (TNH) | Normal appearing thyroid tissue adjacent to TNH | Absent |
| V-NC | NC10 | 38 | Female | 4.52 | 16.7 | 5.79 | 9 | 16.5 | 1.04 | Papillary Thyroid Carcinoma  (PTC) | Normal appearing thyroid tissue adjacent to PTC | Absent |
| V-NC | NC11 | 56 | Female | 4.74 | 14.2 | 2.03 | 31.1 | 18.4 | 1.02 | Papillary Thyroid Carcinoma  (PTC) | Normal appearing thyroid tissue adjacent to PTC | Absent |
| V-NC | NC12 | 53 | Female | 4.97 | 17.6 | 1.4 | 11.8 | 16.3 | 1.29 | Thyroid Nodular Hyperplasia  (TNH) | Normal appearing thyroid tissue adjacent to TNH | Absent |
| V-NC | NC13 | 62 | Female | 4.58 | 17 | 5.06 | 9 | 15.3 | 1.13 | Follicular Thyroid Neoplasm  (FTN) | Normal appearing thyroid tissue adjacent to FTN | Absent |
| V-NC | NC14 | 36 | Female | 5.05 | 17 | 1.15 | 9 | 16.1 | 1.49 | Papillary Thyroid Carcinoma  (PTC) | Normal appearing thyroid tissue adjacent to PTC | Absent |
| V-NC | NC15 | 33 | Female | 5.15 | 18 | 1.57 | 22.9 | 22.6 | 0.8 | Papillary Thyroid Carcinoma  (PTC) | Normal appearing thyroid tissue adjacent to PTC | Absent |
| Disease State:​ The primary histopathological diagnosis from the surgical specimen that led to thyroidectomy (e.g., papillary thyroid carcinoma, chronic lymphocytic thyroiditis). Tissue Type:​ For cases with a focal lesion (e.g., carcinoma, nodule), the sample is “normal appearing thyroid tissue adjacent to the lesion”. For the diffuse disease chronic lymphocytic thyroiditis †, the sampled tissue is designated as “thyroid parenchyma involved by lymphocytic thyroiditis”. Lymphocytic Thyroiditis Status:​ Determined histologically; “Present” for the V-HT group and “Absent” for the V-NC group. †​ For patients with chronic lymphocytic thyroiditis as the primary disease state, the entire gland is involved by the inflammatory process; therefore, the tissue sampled is representative of the diseased parenchyma, not adjacent ‘normal’ tissue.” | | | | | | | | | | | | |
